# The burden and clinical trajectory of immune checkpoint inhibitor-induced endocrinopathies: an 8-year experience

**DOI:** 10.1186/s12916-024-03812-2

**Published:** 2024-12-18

**Authors:** Fateen Ata, Adeel Ahmad Khan, Emad Algorani, Amr Faisal Musaed Alsharafi, Reham Abo Shdid, Mohammad Nofal, Ayman R. Ibrahim, Loai Abdullah, Khalil Youssef El Annan, Tareq Emad Hawash Al-Bkoor, Kakil Ibrahim Rasul, Tarik Elhadd, Haval Surchi

**Affiliations:** 1 Department of Endocrinology, Hamad General Hospital, Hamad Medical Corporation, PO BOX 3050, Doha, Qatar; 2 Department of Internal Medicine, Hamad Medical Corporation, Doha, Qatar; 3 Department of Family Medicine, Hamad Medical Corporation, Doha, Qatar; 4 Department of Geriatric Medicine, Hamad Medical Corporation, Doha, Qatar; 5 Department of Oncology, National Center for Cancer Care and Research, Hamad Medical Corporation, Doha, Qatar

**Keywords:** Immune checkpoint inhibitors, Pembrolizumab, Nivolumab, Atezolizumab, Ipilimumab, Durvalumab, Avelumab, Endocrinopathies

## Abstract

**Background:**

Immune checkpoint inhibitors (ICIs) have revolutionized the management of cancer patients, but the emergence of ICI-related endocrinopathies (IREs) has introduced new clinical challenges. Despite worldwide recognition of these adverse effects, data from the Middle East is scarce.

**Methods:**

This retrospective-observational study included adult cancer patients who received at least one dose of ICI between January 2015 and January 2023. Descriptive statistics and multivariable regression (MVR) models were applied to delineate the incidence and clinical impact of IREs.

**Results:**

The median age of 649 included patients was 55 years, with male preponderance (70.7%). The incidence of IREs was 26.7%, dominated by primary hypothyroidism (62.4%), insulin deficiency (15%), and primary hyperthyroidism (13.9%). Pembrolizumab (62%) was the most utilized ICI among the study cohort, followed by nivolumab (23.7%), atezolizumab (12.5%), durvalumab (0.9%), avelumab (0.6%) and ipilimumab (0.1%). The mortality rates in the cohort and the IRE subgroup were 43.4% and 42.2%. MVR revealed age (OR 1.02, 95% CI (1.003–1.03), *P* = 0.02), pre-ICI white-cell (WBC) count (OR 0.94, 95% CI (0.89–0.99), *P* = 0.04), pembrolizumab (OR 2.6, 95% CI (1.05–6.3), *P* = 0.04), and nivolumab use (OR 2.6, 95% CI (1.04–6.6), *P* = 0.04) as significant predictors of IREs. After MVR, factors influencing mortality in the subgroup with IREs included a higher age (OR 1.1, 95% CI 1.04–1.2, *P* = 0.001) and platelet-to-lymphocyte ratio (OR 1.004, 95% CI 0.7–1.4, *P* = 0.006).

**Conclusions:**

This first extensive Middle Eastern and South Asian cohort reported a higher-than-previously known incidence of IREs. Hypothyroidism, insulin deficiency, and hyperthyroidism were the commonest IREs, with pembrolizumab being the commonest ICI. IRE development was associated with higher age, a low WBC count, pembrolizumab, and nivolumab use. The development of IREs did not seem to influence mortality. Further research on IREs is imperative to optimize management guidelines in the era of precision medicine.

**Supplementary Information:**

The online version contains supplementary material available at 10.1186/s12916-024-03812-2.

## Background

Immune checkpoint inhibitors (ICIs) are monoclonal antibodies first approved by the Food and Drug Administration (FDA) in 2011 for the treatment of melanoma [1]. The various types of ICIs include programmed death-1 (PD-1) inhibitors, programmed death-ligand-1 (PD-L1) inhibitors, and cytotoxic T-lymphocyte antigen 4 (CTLA-4) inhibitors, depending upon their targets in the immune system [2]. Currently, ICIs have revolutionized the therapeutic landscape of cancer, offering targeted treatment for at least 17 types of cancer [3]. Abundant evidence supports the mortality benefits of ICIs in various malignancies [4, 5]. In addition to improving mortality, multiple studies have shown improved quality of life (QOL) among patients with malignancies treated with ICIs [6–8]. The utilization of ICIs for treating malignancies is rapidly evolving with emerging evidence. In the USA alone, since FDA approval, expenses associated with ICI prescriptions have increased from 2.8 million USD to 4.1 billion USD in 2021 [9]. Despite their unprecedented mortality and QOL benefits in the cancer population, ICIs have introduced a spectrum of immune-related adverse effects (irAEs), posing a new clinical challenge in their utilization by increasing patient morbidity and, rarely, mortality [10, 11]. The endocrine system is the most common organ system affected by the immune dysregulatory effects of ICIs [2]. Current data on ICI-related endocrinopathies (IREs) indicate hypothyroidism, insulin deficiency, pituitary hypophysitis, and adrenal insufficiency as the predominant disorders with varying frequencies [12, 13]. The reported incidence of IREs ranges from 0.4 to 20% [2, 11, 14]. In a recently published consensus report (in 2022), the American Association of Clinical Endocrinology (AACE) suggested preemptive screening and vigilant monitoring for IREs and advocated baseline endocrinology assessments, followed by regular monitoring during ICI therapy [11].

Despite the global recognition of IREs, a literature gap remains in the epidemiological data from the Middle East and Asian regions. Moreover, Western cohorts provide robust evidence on the prevalence and management of IREs. Nevertheless, the generalizability of these findings to Middle Eastern and South Asian populations, secondary to genetic diversity and environmental differences, which are known influencers of cancer etiology, clinical behavior, and treatment response, remains unclear [15–17]. Qatar’s diverse patient population and the increasing utilization of ICIs across its healthcare system provide an opportunity to understand the epidemiology, clinical course, and outcomes of IREs in this population. However, to date, data from the region are limited to case reports and small series. However, extensive studies systematically quantifying the incidence of IREs and their impact on patient outcomes are lacking [18, 19]. Our study aims to bridge this critical knowledge gap by providing valuable insights into the epidemiology of IREs and their impact on the oncological population from the Middle East and South Asia.

## Methods

### Study design and participants

This retrospective observational study included adult patients in Qatar who received ICIs for various types of cancer from January 2015 to January 2023.

### Inclusion and exclusion criteria

This study included adult patients (≥ 18 years of age) with various malignancies treated with at least one dose of ICI. Patients were included if they were treated with any ICI (PD-1 inhibitor, PD-L1 inhibitor, or CTLA-4 inhibitor). Patients who were diagnosed with new-onset primary or central hypothyroidism and hyperthyroidism, adrenal insufficiency, insulin deficiency, diabetes insipidus, hypogonadism, growth hormone deficiency, or hypoparathyroidism were included in the post-ICI endocrinopathy group. In contrast, those who did not develop new endocrinopathy were added to the nonendocrinopathy group. Patients who did not receive at least one dose of the scheduled ICI therapy were excluded from the study.

### Data sources and measures

Clinical data were retrieved from electronic medical records (Oracle Cerner®) based on the administration of specific ICI drugs. The data included demographic information, medical history, details of ICI treatment (type and doses), and subsequent endocrine complications. The diagnosis of endocrinopathies was established via endocrinology/medicine clinic progress notes and clinical and biochemical data of the patients (abnormal at least twice). Insulin deficiency was defined on the basis of new insulin requirements (with low C-peptide levels and elevated glutamic acid decarboxylase (GAD) antibodies where available). Primary hypothyroidism was defined by elevated thyroid-stimulating hormone (TSH) levels and low free thyroxine (T4) levels. Central hypothyroidism was defined by inappropriately normal or low TSH levels with low free T4 levels. Primary adrenal insufficiency was defined by low cortisol levels with elevated adrenocorticotropic hormone (ACTH), and secondary adrenal insufficiency was defined by low cortisol levels with inappropriately normal or low ACTH levels. Hypogonadism was defined by low sex hormone levels (testosterone in males, estradiol in females), low or inappropriately normal luteinizing hormone (LH), and follicle-stimulating hormone (FSH) levels. Hypoparathyroidism was defined by low parathyroid hormone (PTH) levels. Baseline and follow-up magnetic resonance imaging (MRI) scans of the pituitary were reviewed to identify and collect data on pituitary hypophysitis or other relevant pathologies. Follow-up biochemical data were collected at two points: 1 year post-ICI initiation and the last follow-up before the study duration. Cancers were categorized according to their region of occurrence. Those with fewer than ten instances were aggregated into the “other” category for analysis. Platelet-to-lymphocyte ratio (PLR) was calculated for the included patients using the formula PLR = Lymphocyte Count (× 10^3^/μL) ÷ Platelet Count (× 10^3^/μL).

### Outcome measures

The primary outcome was the incidence of IREs. The secondary outcomes were clinical differences among patients with and without IREs, factors influencing the development of IREs, and factors influencing mortality in patients who developed IREs.

### Statistical analysis

Descriptive statistics summarized the cohort’s characteristics, with continuous variables presented as the means ± standard deviations (SD) or medians with interquartile ranges (IQRs) based on distribution normality. Categorical variables are presented as frequencies and percentages. Student’s *t* test for continuous variables and the chi-square or Fisher’s exact tests for categorical variables were used to compare groups as appropriate. Associations between ICI treatment and the development of endocrinopathies were explored using multivariable regression (MVR) models adjusted for potential confounders. The MVR models included clinically relevant variables and variables that achieved a *P* value < 0.1 in univariate analysis. A *P* value of < 0.05 indicated statistical significance.

### Ethical considerations

The Institutional Review Board (IRB) of the Hamad Medical Corporation (HMC) approved the study (protocol ID MRC-01–23-001). Due to its retrospective design, informed consent was waived. All patient data were deidentified to maintain confidentiality and compliance with ethical guidelines and regulations. The study adhered to the Declaration of Helsinki.

## Results

### Study cohort

**Fig. 1:**
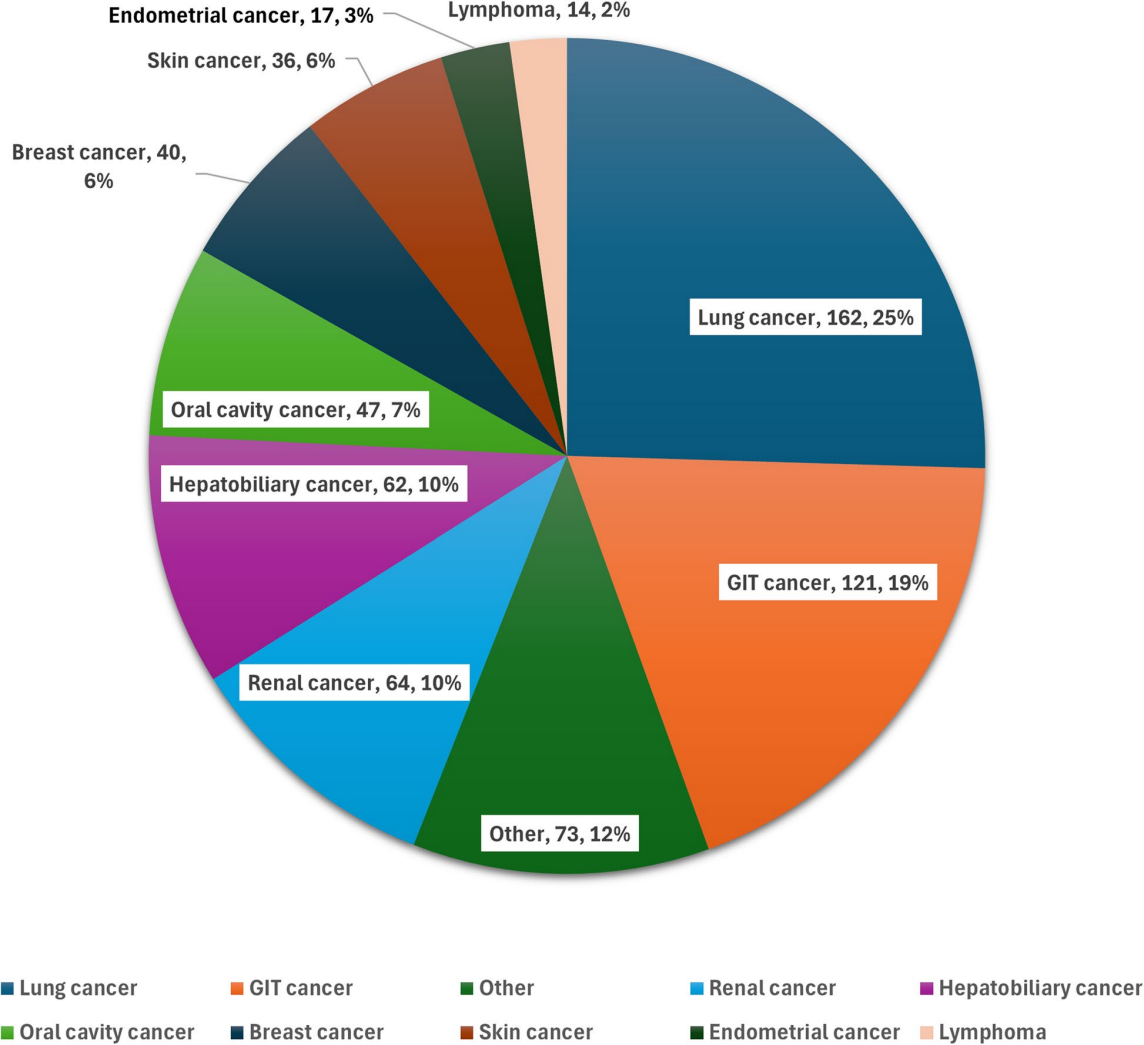
Distribution of primary cancer types among patients receiving immune checkpoint inhibitors. This pie chart illustrates the proportional representation of different cancer diagnoses in our cohort of 649 patients, highlighting the diversity of oncological conditions treated with ICI therapy

**Fig. 2:**
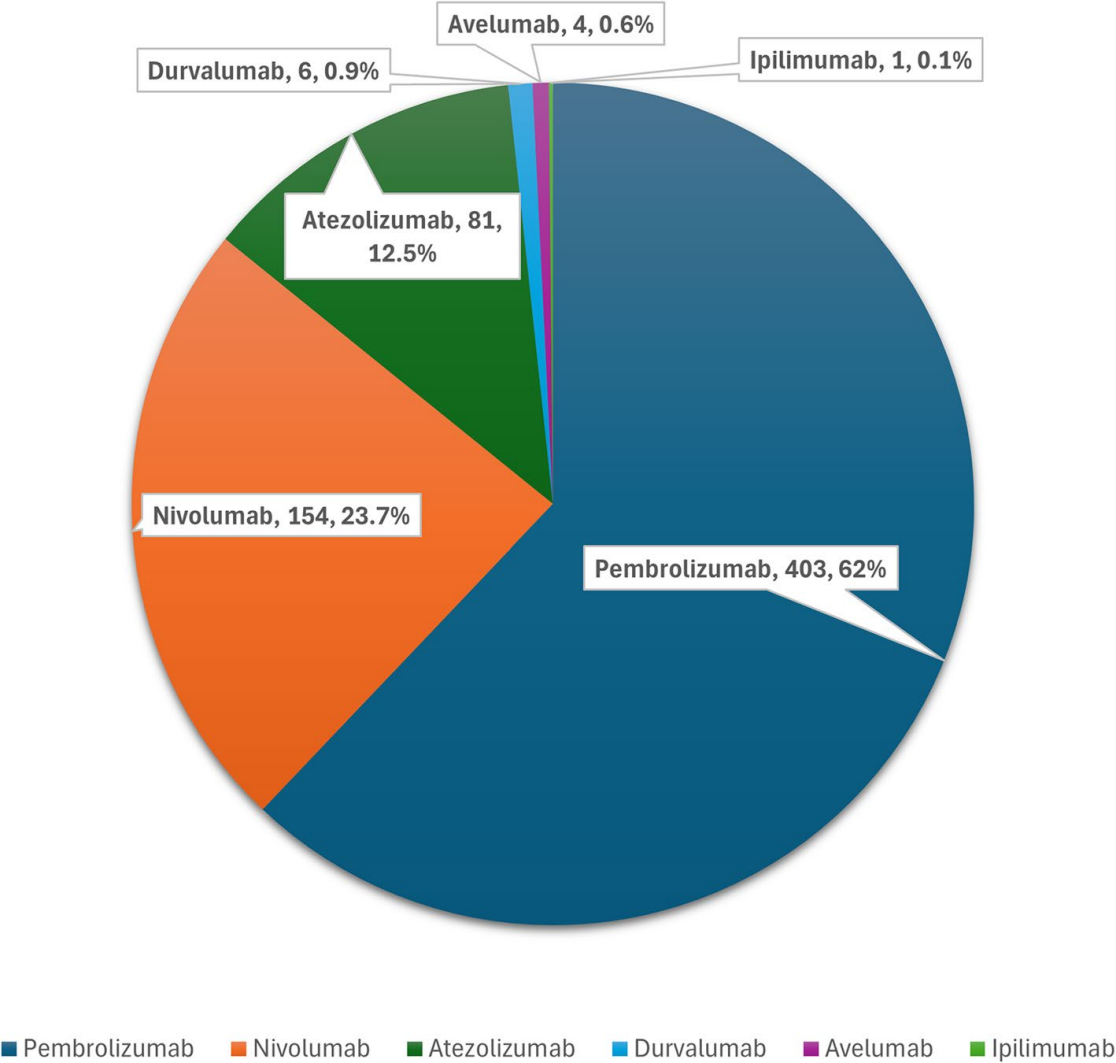
Types of immune checkpoint inhibitors administered to patients throughout the study

During the 8 years of the study, 649 patients received various ICIs. The median age was 55 (45–64) years. The cohort was predominantly composed of 459 males (70.7%). Most participants were of Arab ethnicity (53.5%), followed by South Asians (29.3%). The median baseline body mass index (BMI) was 25.2 (21.7–29.4) kg/m^2^. Preexisting comorbid conditions ranged from type 2 diabetes mellitus (T2DM) being most prevalent (33.1%) to adrenal insufficiency (0.3%). The demographic data are detailed in Table 1. Among the types of malignancies, lung cancer was the most prevalent (25%), and lymphoma was the least reported cancer (2%). Other types of malignancies included gastrointestinal, renal, hepatobiliary, oral cavity, breast, skin, and endometrial cancers and had varying frequencies (Fig. 1). Most patients received pembrolizumab (62%), followed by nivolumab (23.8%), atezolizumab (12.5%), durvalumab (0.9%), avelumab (0.6%), and ipilimumab (0.1%) (Fig. 2).

**Table 1:** Comprehensive demographic, clinical, and treatment profile of patients undergoing ICI therapy. Data are reported as available, reported as the mean (with SD), median (with IQR), and N (with %) as appropriate

**Characteristics**	**Result**
Age, years (*N*=649)	55 (45-64)
Gender (*N*=649)	
Male	459 (70.7%)
Female	190 (29.3%)
Ethnicity (*N*=649)	
Arab	347 (53.5%)
South Asian	190 (29.3%)
Other	81 (12.5%)
White	31 (4.7%)
Baseline BMI (kg/m^2^) (*N*=649)	25.2 (21.7-29.4)
Preexisting Comorbidities (*N*=649)	
T2DM	215 (33.1%)
Hypertension	213 (32.8%)
Dyslipidemia	99 (15.23%)
Chronic Kidney Disease	68 (10.5%)
Ischemic Heart Disease (IHD)	46 (7.08%)
Hypothyroidism	84 (12.9%)
Congestive Heart Failure	21 (3.2%)
Hyperthyroidism	8 (1.23%)
Stroke	16 (2.5%)
T1DM	3 (0.5%)
Hypopituitarism	1 (0.1%)
Adrenal Insufficiency	2 (0.3%)
Baseline MRI head findings (*N*=222)	
Normal pituitary	214 (94.3%)
Likely pituitary microadenoma	1 (0.4%)
Suspected pituitary microadenoma.	1 (0.4%)
Empty Sella	4 (1.8%)
Pituitary gland infundibulum secondary nodular neoplasm	1 (0.4%)
Pituitary gland metastasis	1 (0.4%)
Number of doses of ICI given in total	5 (2-12)
Radiotherapy details (N=266)	
Head and neck.	98 (36.8%)
Head and neck + other body parts.	1 (0.4%)
Other body parts	167 (62.8%)

The BMIs were as follows: underweight (<18.5 kg/m²), normal (18.5–24.9 kg/m²), overweight (25–29.9 kg/m²), and obese (≥30 kg/m²)

*BMI* Body mass index, *T2DM* Type 2 diabetes mellitus, *IHD* Ischemic heart disease, *MRI* Magnetic resonance imaging, *ICI* Immune checkpoint inhibitor

### Prevalence of endocrinopathies

**Fig. 3:**
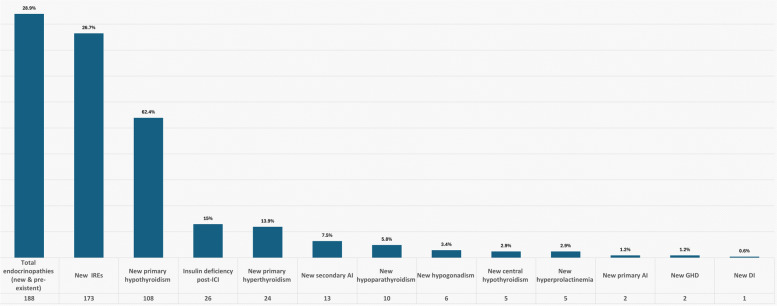
Bar graph delineating the incidence of various endocrinopathies following immune checkpoint inhibitor therapy

**Fig. 4:**
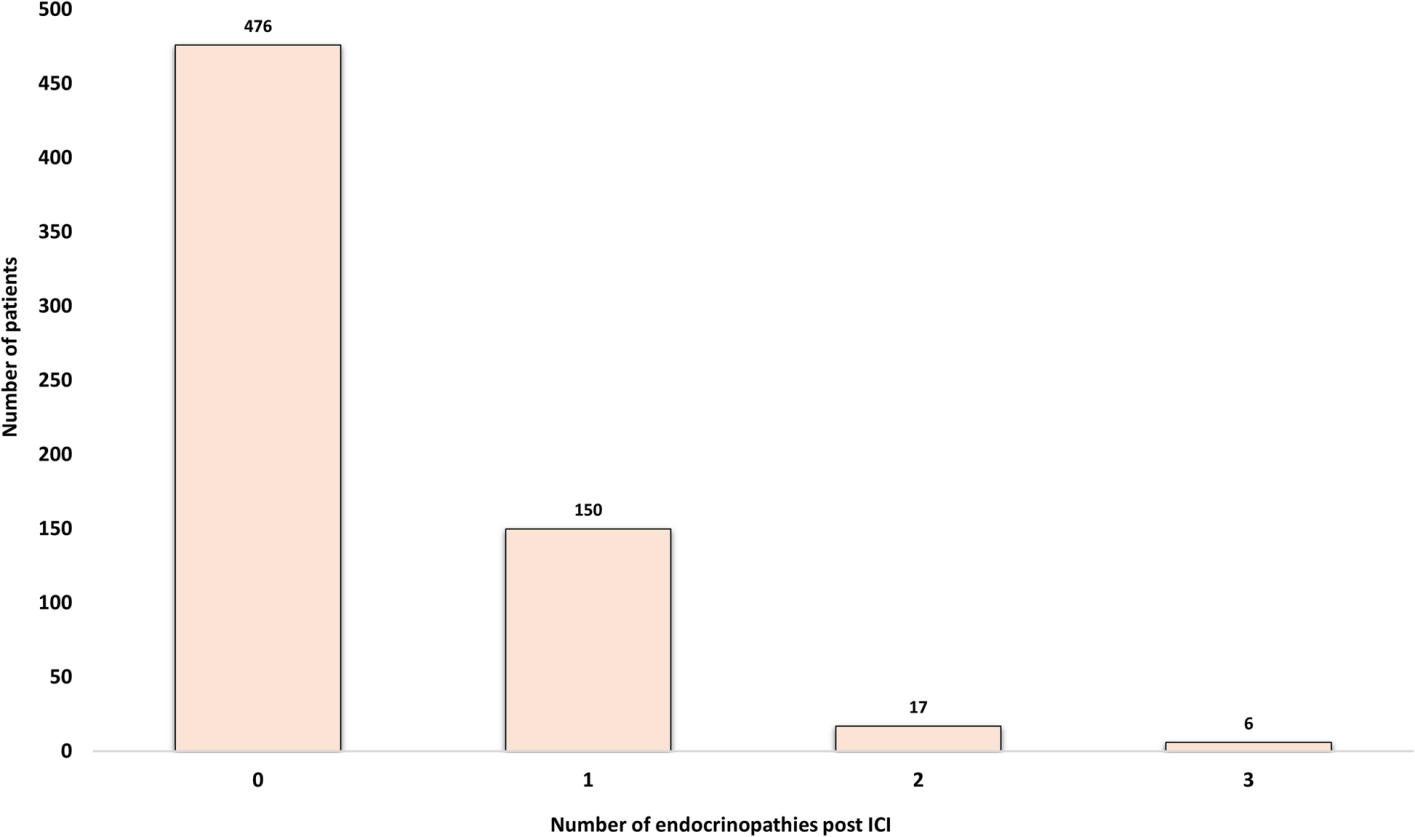
Number of patients categorized by the total number of IREs

In our cohort, the prevalence of any endocrinopathy (new and preexisting combined) post-ICI was 29% (188 patients), while the incidence of new IREs was 26.7% (173 patients). Among the various reported IREs, new-onset primary hypothyroidism was most prevalent (62.4%), followed by insulin deficiency post-ICI therapy (15%), new-onset primary hyperthyroidism (13.9%), and secondary adrenal insufficiency (7.5%). Hypothyroidism was most commonly caused by pembrolizumab compared to other ICIs (*P* = 0.01). New diabetes insipidus (DI) was the least documented IRE (0.6%) (Fig. 3). Post-ICI therapy, diabetic ketoacidosis (DKA) was noted in 0.8% (5 out of 649) of patients, 80% (4 out of 5) of whom had new-onset diabetes, and 20% (1 out of 5) of whom had preexisting diabetes. Figure 4 shows the burden of endocrinopathies in the cohort, with the majority (150 patients) presenting with a single IRE. The frequencies of the three commonest IREs divided by the ICI types (programmed death-1 inhibitor (PD-1i) and programmed death-ligand 1 inhibitor (PD-L1i)) are summarized in Table 2.

**Table 2:** Association of ICIS with the three commonest endocrinopathies in the study cohort

Endocrinopathy	PD-1, *N* = 557	PD-L1, *N* = 91
Hypothyroidism developed (*N* = 108)	95 (17.1%)	13 (14.3%)
Hypothyroidism not developed (*N* = 540)	462 (82.9%)	78 (85.7%)
Endocrinopathy	**PD-1, *N* = 557**	**PD-L1, *N* = 91**
Insulin deficiency (*N* = 26)	22 (3.9%)	4 (4.4%)
Insulin deficiency not developed (*N* = 622)	535 (96%)	87 (95.6%)
Endocrinopathy	**PD-1, *N* = 557**	**PD-L1, *N* = 91**
Hyperthyroidism (*N* = 24)	20 (3.6%)	3 (3.3%)
Hyperthyroidism not developed (*N* = 624)	537 (96.4%)	88 (96.7%)

*ICI* Immune checkpoint inhibitor, *PD-L1* Programmed death-ligand 1, *PD-1* Programmed death 1

### Biochemical results in the study cohort

Additional file 1: Table S1 summarizes the biochemical investigations performed before initiating ICI therapy. The median creatinine level was 70 μmol/L (56–90), the median HbA1c level was 5.85% (5.4–6.9), the white blood cell count was 6.9 × 10^3^/L (5–9.3), the hemoglobin level was 11.42 g/dL, and the neutrophil count was 6.3 × 10^3^/L (3.7–53), with a median neutrophil-to-lymphocyte ratio of 3.28 (2.05–5.72). Thyroid function tests (TFTs) revealed a median thyroid-stimulating hormone (TSH) level of 1.81 mIU/L (1.07–3.35), a free T3 level of 4.09 pmol/L, and a free T4 level of 14.9 pmol/L (12.9–16.71). The median morning cortisol level was 349.44 nmol/L, and the median adrenocorticotropic hormone (ACTH) level was 9.3 pg/mL (5–131).

The post-ICI therapy biochemical results are summarized in Additional file 1: Table S2. Thyroid peroxidase (TPO) antibodies were newly positive in 7 out of 36 patients with available data, with 5.6% (2 out of 36) already positive pre-ICIs. The median values for TFTs were as follows: TSH 2.4 mIU/L (1.2–6.7), free T3 4.2 pmol/L (3.1–4.9), and free T4 14.4 pmol/L (11.9–16.6). The mean morning cortisol and median ACTH levels were 340 nmol/L ± 215.8 and 13.9 pg/mL (7.3–38.50), respectively. The median prolactin concentration was 284 mIU/L (203–484). Hypophysitis post-ICI was detected in only one patient via MRI. The median HbA1c and C-peptide levels were 5.9% (5.4–6.8) and 0.57 ng/mL (0.3–2.2), respectively. Additional file 1: Table S3 summarizes the cohort’s biochemical data at the end of the study.

### Comparative analysis of IRE profiles

Among the patients who developed IREs (26.7%) versus those without (73.3%), significant differences were observed (Table 2). The mean age was lower in the IRE group (53.3 ± 13.2 years) than in the non-IRE group (56.9 ± 12.9 years, *P* = 0.001). Arab ethnicity was more prevalent in the IRE group than in the non-IRE group (59.5% vs. 51.3%; *P* = 0.02). On the other hand, whites were more prevalent in the non-IRE group than in the IRE group (5.9% vs. 1.7%; *P* = 0.02). The baseline body mass index (BMI) was greater in patients with new IREs (26.6 kg/m^2^ vs. 24.8 kg/m^2^, *P* = 0.001). The most common comorbid conditions were detected in the IRE group; these included hypertension (42.8% vs. 29.2%, *P* = 0.001), dyslipidemia (20.8% vs. 13.2%, *P* = 0.018), chronic kidney disease (CKD) (15.6% vs. 8.6%, *P* = 0.01), and ischemic heart disease (10.4% vs. 5.8%, *P* = 0.04). According to the pre-ICI biochemical data, the median serum creatinine level was greater in the IRE group than in the non-IRE group (74 μmol/L vs. 68.5 μmol/L, *P* = 0.01). In contrast, the neutrophil-to-lymphocyte ratio (NLR) was lower in patients with new IREs (2.4 vs. 3.4, *P* = 0.002). Pembrolizumab was the most commonly used ICI in both groups (60.7% in the IRE group and 62.5% in the non-IRE group). Durvalumab was more commonly used in the IRE group (2.3% vs 0.4%), whereas atezolizumab was more commonly used in the non-IRE group (13.9% vs. 8.7%) (*P* = 0.008). Furthermore, patients in the IRE group reported a greater median number of ICI doses (9 vs. 4, *P* < 0.001). A greater proportion of patients in the IRE group received radiotherapy, although the difference was not statistically significant (45.6% vs. 39.2%; *P* = 0.1). There were no significant differences in the development of IREs among patients when categorized into < 60 years and ≥ 60 years old, except mortality which was higher in ≥ 60 years group (39.9% in < 60 vs. 49.7% in ≥ 60 years group, *P* = 0.01) (Additional file 1: Table S4).

### Comparative analysis of ICI profiles

Among the subgroup of patients who developed IREs (*n* = 173), a comparative analysis was performed between patients taking PD-1 inhibitors (pembrolizumab and nivolumab) and patients taking PD-L1 inhibitors (atezolizumab, durvalumab, and avelumab) (Table 3). Age, sex, ethnicity, preexisting endocrinopathies, number of ICI doses, number of IREs developed per person, number of new IREs, and mortality were not different between the two groups (Additional file 1: Table S5).

**Table 3:** Differences in the clinical and biochemical profiles between patients with and without new endocrinopathies after ICI therapy. Data are reported as available and reported as the mean (with SD), median (with IQR), and N (with %) as appropriate

**Parameter**	**IRE** **(*N*=173, 26.7%)**	**No IRE** **(*N*= 476, 73.3%)**	***P* Value**
Age	53.3 ± 13.2	56.9 ± 12.9	**0.001**
Gender			
Male	122 (70.5%)	337 (70.8%)	0.9
Female	51 (29.5%)	139 (29.2%)	
Ethnicity			
Arab	103 (59.5%)	244 (51.3%)	
South Asian	52 (30.1%)	138 (29%)	**0.02**
Other	15 (8.7%)	66 (13.9%)	
White	3 (1.7%)	28 (5.9%)	
Baseline BMI (kg/m^2^)	26.6 (23-31)	24.8 (21.6-28.5)	**0.001**
Preexisting Comorbidities			
T2DM	63 (36.4%)	152 (31.9%)	0.2
Hypertension	74 (42.8%)	139 (29.2%)	**0.001**
Dyslipidemia	36 (20.8%)	63 (13.2%)	**0.018**
Chronic Kidney Disease	27 (15.6%)	41 (8.6%)	**0.01**
Ischemic Heart Disease (IHD)	18 (10.4%)	28 (5.8%)	**0.04**
Hypothyroidism	13 (7.5%)	71 (14.9%)	**0.01**
Congestive Heart Failure	7 (4.1%)	17 (16.2%)	0.7
Hyperthyroidism	1 (0.6%)	7 (1.5%)	0.6
Stroke	3 (1.7%)	13 (2.7%)	0.5
T1DM	0	3 (0.6%)	1
Hypopituitarism	0	1 (0.2%)	1
Adrenal Insufficiency	0	2 (0.4%)	1
HBA1C %	5.9 (5.5-7)	5.8 (5.4-6.8)	0.2
Creatinine, μmol/L	74 (59-93)	68.5 (55-88)	**0.01**
PLR	145.3 (64.2-229.2)	143.9 (27-259.6)	0.7
NLR	2.4 (1.6-4.9)	3.4 (2.2-6.2)	**0.002**
Sodium, mmol/L	138 (136-140)	138 (134-140)	**0.05**
Potassium, mmol/L	4.3 ± 0.5	4.3 ± 0.5	0.4
Fasting Glucose, mmol/L	5.6 (5.1-6.9)	6 (5-7.7)	0.3
Random Glucose, mmol/L	6.6 (4.9-8.9)	6.7 (5.6-9)	0.1
WBC, ×10^3^/L	6.6 (5.6-9)	7 (5.1-9.6)	0.9
Hemoglobin, g/dL	11.44 ± 2	11.41 ± 2	0.9
TSH, mIU/L	1.8 (1.1-3.1)	1.9 (1-3.7)	0.6
Free T3, pmol/L	3.9 ± 1	4.2 ± 1.2	0.2
Free T4, pmol/L	14.6 ± 3.2	15.1 ± 4.3	0.2
Radiotherapy details	(*N*=79, 45.6%)	(*N*=187, 39.2%)	
Head and neck.	24 (30.4%)	74 (39.6%)	
Head and neck + other body parts.	1 (1.3%)	0	0.1
Other body parts	54 (68.3%)	113 (60.4%)	
ICI drug			
Atezolizumab	15 (8.7%)	66 (13.9%)	
Avelumab	3 (1.7%)	1 (0.2%)	
Durvalumab	4 (2.3%)	2 (0.4%)	
Ipilimumab	1 (0.5%)	0	**0.008**
Nivolumab	45 (26%)	109 (23%)	
Pembrolizumab	105 (60.7%)	297 (62.5%)	
Type of malignancy	*N*=169	*N*=467	
Lung cancer	46 (27.2%)	116 (24.8%)	
Git cancer	33 (19.5%)	88 (18.8%)	0.7
Other	19 (11.2%)	54 (11.6%)	
Renal cancer	16 (9.4%)	48 (10.2%)	
Hepatobiliary cancer	13 (7.7%)	49 (10.5%)	
Oral cavity cancer	14 (8.3%)	33 (7.1%)	
Breast cancer	13 (9%)	27 (5.5%)	
Skin cancer	6 (3.6%)	30 (6.4%)	
Endometrial cancer	3 (1.8%)	14 (3%)	
Lymphoma	4 (2.4%)	10 (2.1%)	
Number of ICI doses	9 (4-17)	4 (2-9)	**< 0.001**
Mortality	73 (42.2%)	209 (44%)	0.7

The normal ranges used were as follows: creatinine, 62-106 μmol/L; urea, 2.5-7.8 mmol/L; sodium, 133-146 mmol/L; potassium, 3.5-5.3 mmol/L; fasting glucose, <11.1 mmol/L; random glucose, 3.3-5.5 mmol/L; HbA1c, <5.7%; WBC, 4-10 ×103/L; hemoglobin, 12-15 g/dL; neutrophil, 2-7 ×103/L; platelets, 150-410 ×103/L; lymphocyte count, 1-3 ×103/L; TSH, 0.3-4.2 mIU/L; free T3, 3.7-6.4 pmol/L; and free T4, 11-23.3 pmol/L. *P* values in bold indicate statistical significance

*ICI* Immune checkpoint inhibitor, *IRE* Immune checkpoint inhibitor-related endocrinopathy, *SD* Standard deviation, *IQR* Interquartile range, *T2DM* Type 2 diabetes mellitus, *IDDM* Insulin-dependent diabetes mellitus, *NLR* Neutrophil-to-lymphocyte ratio, *WBC* White blood cell, *TSH* Thyroid stimulating hormone, *FSH* Follicle stimulating hormone, *LH* Luteinizing hormone, *TRAB* TSH receptor antibody, *TPO* Thyroid peroxidase, *IGF-1* Insulin-like growth factor 1, *HbA1c* Hemoglobin A1c, *DKA* Diabetic ketoacidosis

### Factors influencing the development of IREs

Univariate (UV) analysis followed by MVR analysis was performed on patients who developed new IREs (*n* = 173) to identify factors affecting endocrinopathy development post-ICI therapy (Table 4). According to the univariate analysis, factors that increased the risk of developing IREs included age at ICI initiation (odds ratio [OR] 1.02, 95% CI = 1–1.03, *P* = 0.003), initial BMI (OR = 1.04, 95% CI = 1.01–1.07, *P* = 0.03), and CKD (OR = 1.9, 95% CI = 1.1–3.2, *P* = 0.01). Additionally, the serum creatinine and serum sodium levels were associated with IRE risk (OR 1.003, 95% CI: 1–1.006, *P* = 0.02 for creatinine; OR 1.05, 95% CI: 1–1.1, *P* = 0.02 for sodium), as was the serum WBC count (OR 0.9, 95% CI: 0.8–0.9, *P* = 0.01). The use of avelumab (OR 13.2, 95% CI: 1.3–135.8, *P* = 0.03) and durvalumab (OR 8.8, 95% CI: 1.5–52.6, *P* = 0.02) significantly increased the likelihood of developing an endocrinopathy in the UV analysis when compared to that of atezolizumab. Finally, the number of ICI doses and cumulative ICI doses were significant predictors of endocrinopathy development post-ICI therapy (OR 1.03, 95% CI = 1.02–1.05, *P* < 0.001; OR 1.0006, 95% CI = 100,002–100009, *P* < 0.001, respectively). In the subsequent MVR analysis (adjusted for various clinical and demographic factors), age at ICI initiation (OR 1.02, 95% CI = 1.003–1.03; *P* = 0.02), pre-ICI serum WBC (OR = 0.94, 95% CI = 0.89–0.99, *P* = 0.04), nivolumab use (OR = 2.6, 95% CI = 1.04–6.6, *P* = 0.04), and pembrolizumab use (OR = 2.6, 95% CI = 1.05–6.3, *P* = 0.04) were the significant factors that predicted the development of endocrinopathy.

**Table 4:** Univariate and multivariate logistic regression analysis of factors influencing the development of endocrinopathy following immune checkpoint inhibitor therapy

**Variable**	**Univariate Analysis**		**Multivariate Analysis**	
	**Odds Ratio (95% CI)**	***P* value**	**Odds Ratio (95% CI)**	***P* value**
Age (years) at ICI initiation	1.02 (1-1.03)	**0.003**	1.02 (1.003-1.03)	**0.02**
Male Gender	0.9 (0.6-1.4)	0.8		
Ethnicity (compared to Arab)				
South Asian	0.9 (0.6-1.3)	0.57		
Others	0.5 (0.3-0.99)	**0.04**		
White	0.3 (0.1-1)	0.05		
Initial BMI (kg/m^2^)	1.04 (1.01-1.07)	**0.03**		
CKD	1.9 (1.1-3.2)	**0.01**		
Pre-ICI-biochemical tests				
Serum creatinine, μmol/L	1.003 (1-1.006)	**0.02**		
Serum sodium, mmol/L	1.05 (1-1.1)	**0.02**		
Serum WBC ×10^3^/L	0.9 (0.8-0.9)	**0.01**	0.94 (0.89-0.99)	**0.04**
Type of cancer (compared to Lymphomas)				
Breast cancer	1.5 (0.4-5.6)	0.5		
Endometrial cancer	0.5 (0.1-2.9)	0.4		
GIT cancer	0.9 (0.2-3.1)	0.9		
Hepatobiliary cancer	0.7 (0.2-2.7)	0.6		
Lung cancer	0.99 (0.3-3.3)	0.9		
Oral cavity cancer	1.06 (0.2-3.9)	0.9		
Renal cancer	0.8 (0.2-3)	0.7		
Skin cancer	0.5 (0.1-2.1)	0.3		
Other cancer types	0.8 (0.2-3.1)	0.8		
Type of ICI (compared to Atezolizumab)				
Avelumab	13.2 (1.3-135.8)	**0.03**		
Durvalumab	8.8 (1.5-52.6)	**0.02**		
Nivolumab	1.8 (0.9-3.6)	0.06	2.6 (1.04-6.6)	**0.04**
Pembrolizumab	1.6 (0.8-2.8)	0.1	2.6 (1.05-6.3)	**0.04**
Number of ICI doses	1.03 (1.02-1.05)	**< 0.001**		

The normal concentrations were as follows: creatinine 62-106 μmol/L, sodium 133-146 mmol/L, and white blood cell (WBC) count 4-10 ×103/L. *P* values in bold indicate statistical significance

*P* value for the multivariate logistic regression model: <0.001. The model was adjusted for ethnicity, sex, CKD status, pre-ICI creatinine concentration, pre-ICI sodium concentration, cancer type (breast, endometrial, GIT, hepatobiliary, oral cavity, renal, skin, or other), ICI treatment (durvalumab), number of ICI doses, and cumulative ICI doses

*ICI* Immune checkpoint inhibitor, *CI* Confidence interval, *BMI* Body mass index, *CKD* Chronic kidney disease, *WBC* White blood cell, *GIT* Gastrointestinal tract

### Factors influencing mortality among patients who developed endocrinopathy post-ICI

Univariate (UV) and multivariate ratio (MVR) analyses were used to evaluate the factors influencing mortality following IREs (Table 5). Factors that were significant according to the UV analysis included age at ICI initiation (OR 1.04, 95% CI: 1.02–1.07, *P* < 0.001), serum sodium levels (OR 0.9, 95% CI: 0.8–0.9, *P* = 0.01), HbA1c percentage (OR 1.3, 95% CI: 1.01–1.6, *P* = 0.03), hemoglobin levels (OR 0.8, 95% CI: 0.7–1, *P* = 0.03), platelet-to-lymphocyte ratio (PLR) (OR 1.001, 95% CI: 1.0001–1.003, *P* = 0.03), the number of ICI doses (OR 0.9, 95% CI: 0.91–0.98, *P* = 0.001), the presence of two IREs compared to one (OR 0.2, 95% CI: 0.07–0.9, *P* = 0.04), and the total cumulative number of ICI doses (OR 0.99, 95% CI: 0.998–0.999, *P* = 0.02). According to the MVR analysis, only age at ICI initiation (OR 1.1, 95% CI: 1.04–1.2; *P* = 0.001) and PLR (OR 1.004, 95% CI: 0.7–1.4; *P* = 0.006) were found to be statistically significant factors for predicting mortality.

**Table 5:** Univariate and multivariate logistic regression analysis of factors influencing mortality following endocrinopathies post immune checkpoint inhibitor therapy

**Variable**	**Univariate Analysis**		**Multivariate Analysis**	
	**Odds Ratio (95% CI)**	***P* value**	**Odds Ratio (95% CI)**	***P* value**
Age (years) at ICI initiation	1.04 (1.02-1.07)	**<0.001**	1.1 (1.04-1.2)	**0.001**
Male Gender	1.5 (0.8-3)	0.2		
Ethnicity (compared to Arab)				
South Asian	0.9 (0.4-1.7)	0.7		
Others	0.6 (0.2-2)	0.4		
White	2.6 (0.2-29.3)	0.4		
Preexisting T2DM	1.7 (0.9-3.2)	0.08		
Pre-ICI-biochemical tests				
Serum sodium (mmol)	0.9 (0.8-0.9)	**0.01**		
Random glucose (mmol/L)	1.08 (0.9-1.2)	0.08		
HBA1C (%)	1.3 (1.01-1.6)	**0.03**		
Hemoglobin, g/dL	0.8 (0.7-1)	**0.03**		
PLR	1.001 (1.0001-1.003)	**0.03**	1.004 (0.7-1.4)	**0.006**
Type of ICI (compared to Atezolizumab)				
Avelumab	0.6 (0.04-7.7)	0.6		
Durvalumab	0.4 (0.03-4.5)	0.4		
Nivolumab	1.9 (0.6-6.1)	0.2		
Pembrolizumab	0.6 (0.2-1.8)	0.3		
Number of ICI doses	0.9 (0.91-0.98)	**0.001**		
Number of endocrinopathies post-ICI (compared to 1 endocrinopathy)				
2	0.2 (0.07-0.9)	0.04		
3	1.2 (0.2-6.3)	0.7		
Type of new endocrinopathy post-ICI				
Primary hypothyroidism	0.6 (0.3-1)	0.07		
Insulin deficiency	2 (0.9-4.9)	0.08		
Hypoparathyroidism	3.4 (0.8-13.7)	0.08		
First FU labs post Endocrinopathy				
HbA1c %	1.2 (0.9-1.5)	0.08		
Fasting Glucose, mmol/L	1.1 (1.05-1.2)	**0.002**		
Random Glucose, mmol/L	1.07 (1.01-1.1)	**0.009**		

The inclusion criteria for individuals were as follows: sodium 133-146 mmol/L, fasting glucose <11.1 mmol/L, random glucose 3.3-5.5 mmol/L, HbA1c <5.7%, and hemoglobin 12-15 g/dL. P values in bold indicate statistical significance

*P* value <0.001. The model was adjusted for ethnicity, sex, preexisting type 2 diabetes status, pre-ICI sodium concentration, pre-ICI HBA1C concentration, pre-ICI hemoglobin level, type of ICI, number of ICI doses, cumulative dose of ICI (total and until the development of ICI), number of endocrinopathies, post-ICI new hypothyroidism, post-ICI new insulin deficiency, post-ICI new hypoparathyroidism, post-ICI HBA1C, post-ICI fasting and random blood glucose

*ICI* Immune checkpoint inhibitor, *T2DM* Type 2 diabetes mellitus, *PLR* Platelet-to-lymphocyte ratio, *FU* Follow-up

## Discussion

In this large regional cohort of patients treated with ICIs, the incidence of IREs was 26.7%. Lung cancer was the most common malignancy type in this cohort (25%). The ICIs studied in the study population included PD-1 inhibitors (pembrolizumab (62%) and nivolumab (23.7%), respectively), PD-L1 inhibitors (atezolizumab (12.5%), durvalumab (0.9%), and avelumab (0.6%), and CTLA-4 inhibitors (ipilimumab (0.1%)). New-onset primary hypothyroidism was most prevalent (62.4%), followed by insulin deficiency (15%) and primary hyperthyroidism (13.9%). Most patients had a single IRE (86.7%), 9.8% had two IREs, and 3.4% had three IREs. Many demographic and clinical differences were noted between patients who developed IREs and those who did not. Patients who developed IREs were older, had a higher BMI, were predominantly Arab and South Asian, had a greater comorbidity burden (including HTN, T2D, CKD, IHD, and hypothyroidism), and received a greater number of doses of ICIs during the treatment course. Pembrolizumab and nivolumab exhibited stronger associations with IREs than did the other ICIs in this cohort. Cancer types and mortality did not differ between the two groups. According to the MVR analysis, older age at ICI initiation and type of ICI (nivolumab or pembrolizumab) predicted IRE development. Similarly, mortality was predicted by age at ICI initiation and a higher pre-ICI PLR. The incidence of IRE in this study was higher than that reported in global cohorts, suggesting possible regional variation in susceptibility to IREs [11]. These results form the basis of the epidemiology and outcomes of IREs in the Middle East.

One of the possible reasons for the higher incidence of IRE in the current cohort is the genetic predisposition to autoimmune conditions, which can differ across ethnic populations and hence influence the development of irAEs, including irAEs secondary to ICI therapy [11]. Furthermore, the incidence of IREs in our cohort was similar to that reported by Al Qahtani et al. in a Saudi cohort of 125 patients (20.8%) [18]. Additionally, the prevalence of preexisting comorbid conditions, such as T2DM and hypothyroidism, among others, in our cohort suggested a baseline vulnerability to endocrine dysfunctions. Variabilities in the types of malignancies in the cohorts studied can also contribute to differences in the incidences of IREs. Patients in our cohort had lung cancer, which is one of the most common indications for ICI therapy in the current era and has also been reported to have a higher incidence of irAEs [20, 21]. In contrast, one study in which melanoma was the predominant cancer (64.7%) had only 3.5% IREs, indicating that cancer type may influence the incidence of IREs in these cohorts [22]. Finally, the longer duration of our study may have aided in capturing a greater incidence of IREs.

Pembrolizumab emerged as the most common ICI associated with IREs, consistent with its broad application across malignancies and potent immunomodulatory effects [23]. The occurrence of hypothyroidism, followed by insulin deficiency and hyperthyroidism, highlights the vulnerability of the thyroid axis to the immune modulatory effects of ICIs. The pattern found in our cohort resonates with the literature, in which thyroid dysfunctions are among the most common IREs, highlighting the imperative for proactive monitoring of thyroid function during ICI therapy [2]. Older age and ICI therapy (nivolumab or pembrolizumab) were found to be predictors of IRE development in our cohort. These factors help identify the groups most vulnerable to IRE development. However, studies rigorously investigating risk factors for IREs are scarce. Eun et al. reported that a higher BMI and multiple cycles of pembrolizumab were associated with a greater risk of developing irAEs (including IREs). In contrast, an NLR > 3 was associated with a lower risk of irAEs [24]. The NLR and PLR are evolving prognostic markers for various malignancies, and systemic inflammation and immune response status are correlated with patient outcomes [25]. These biomarkers are attracting interest because they are inexpensive and readily available [26]. However, in the present study, neither the NLR nor the PLR predicted IRE development in the UV or MVR models. The current study revealed a lower risk of developing IREs with a higher WBC count (OR = 0.94), suggesting a complex interplay between systemic inflammatory markers and the incidence of IREs. One study explored the link between eosinophils and the development of post-ICI adrenal insufficiency and reported a positive correlation [27]. Further research into these markers from the perspective of IRE development can enhance our understanding of the pathophysiology and improve patient management by identifying those at greater risk.

ICI-related pituitary hypophysitis is a commonly reported IRE [11]. However, in our study, only one patient had hypophysitis post-ICI. This could be multifactorial, given the differences in cohort and treatment protocols. First, ICI-related pituitary hypophysitis is mainly reported to be secondary to CTLA-4 inhibitors such as ipilimumab, and the use of only one patient on a specific ICI in our cohort may have limited the identification of hypophysitis. In previous studies, variable incidence rates have been reported, depending upon the type and dose of the ICI and study design (retrospective or prospective), among other factors [22, 28–31]. Another reason is the variation in diagnostic approaches, including the underutilization of targeted pituitary imaging at symptom onset (the cornerstone in diagnosing hypophysitis). The clinical presentation of hypophysitis can be subtle and easily overlooked, given its nonspecific symptoms (such as headache and fatigue) amid ongoing malignancy. Nevertheless, immunologic and geographic differences might also play a role in the variable results in our cohort. A study from Saudia with a similar but much smaller cohort also reported a minute percentage of patients developing hypophysitis post-ICI (1.6%) [18].

Like in most previous studies, in the present study, IREs were mostly permanent (approximately 67% of cases) [11, 28, 32, 33]. The persistence of IREs poses a significant clinical challenge for patients already facing physical and psychological challenges from cancer, chemotherapy effects, and a decline in QOL. Studies have reported an association between IREs and reduced mortality. We also studied the effect of IRE development on mortality in our cohort but failed to find significant differences in mortality among those who developed IREs (42.2%) and those who did not (44%) (*P* = 0.7). Previous cohorts that reported a significant reduction in mortality with IRE development were considerably smaller (94 patients compared to 649 patients in our cohort) [34].

**Fig. 5:**
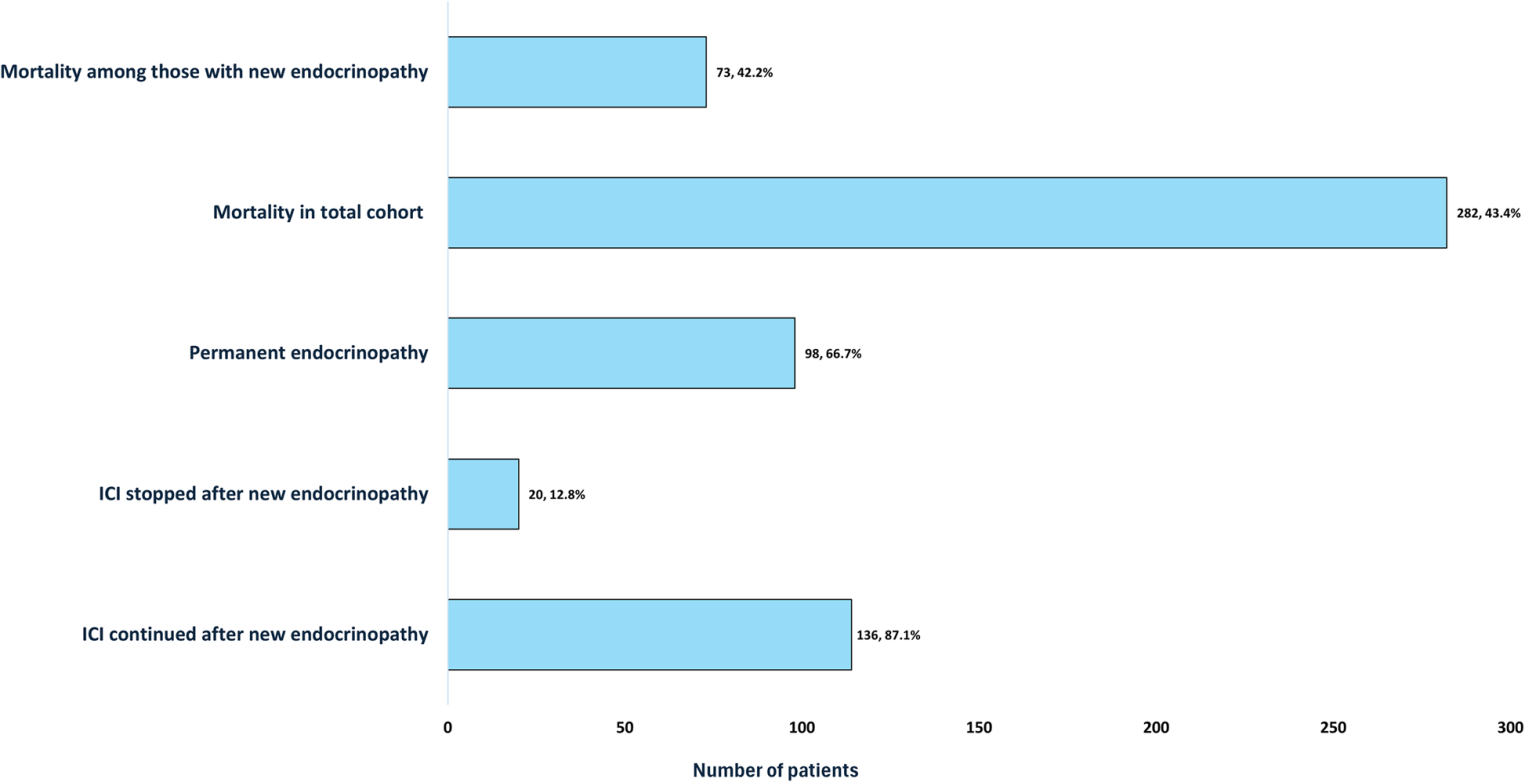
The spectrum of clinical consequences beyond the incidence of IREs, including ICI discontinuation and mortality, highlighting the multifaceted impact of ICIs on patient trajectories

This retrospective study has several notable strengths, including its extensive duration and the review of a spectrum of IREs within a well-defined and diverse patient population (Fig. 5). The longitudinal nature of the study, with a relatively larger sample size from the region, offered insightful information on the patterns and risk factors associated with the development and clinically significant outcome of IREs (such as mortality) via robust regression analyses. Nevertheless, the present work has several limitations. First, the very nature of the design (retrospective) implies that inherent biases, such as selection bias, might be difficult to avoid. The analyses could not adjust for visible confounders (other contaminating chemotherapy regimens) or invisible confounders. Additionally, patient enrollment from a single center may limit the generalizability of the findings to broader populations. A limitation of our comparative analysis of ICI groups (PDL-1 and PD-1 inhibitors) is the notable difference in group sizes, possibly influencing the statistical significance of our findings. Despite the acknowledged limitations, this study is a valuable contribution in light of the expanding evidence on the safety profile of ICIs and their risk of IREs. The current work highlights the importance of strategic monitoring and management of IREs in oncological patients treated with ICIs.

## Conclusions

A higher incidence of IREs than previously known was reported in this first extensive Middle Eastern and South Asian cohort. Hypothyroidism, insulin deficiency, and hyperthyroidism were the most common IREs in our cohort, and pembrolizumab (a PD-1 inhibitor) was the most common ICI used. Pembrolizumab (a PD-1 inhibitor) and nivolumab (a PD-1 inhibitor) were strongly associated with IRE development, and older age and a low WBC count were among the other predictors. The development of IREs did not seem to influence mortality among patients taking ICIs. Further research on IREs is imperative for optimizing management guidelines in the era of precision medicine.

## Supplementary Information

Additional file 1: Table S1. Comprehensive pre-treatment biochemical profile of patients undergoing ICI therapy. Table S2. Dynamic changes in biochemical markers and radiological findings following ICI treatment. Table S3. End-of-study biochemical endocrine profile in patients following ICI therapy. Table S4. Clinical Characteristics of Patients Stratified by Age. Table S5. Comparison of clinical outcomes between PDL1 and PD1 inhibitors in the study population.

## Data Availability

Data sharing requires permission from the Ministry of Public Health, Qatar. Any request for datasets can be made to the Medical Research Center (MRC) Qatar at Hamad Medical Corporation, which will seek legal permission from the MOPH before data sharing. MRChelpdesk@hamad.qa. The corresponding author, Fateen Ata, can be contacted at Fata@hamad.qa to initiate a data availability request.

## Abbreviations

ICIs: Immune checkpoint inhibitors
IREs: ICI-related endocrinopathies
UV: Univariate
MVR: Multivariate regression
OR: Odds ratio
CI: Confidence interval
WBC: White blood cell
QOL: Quality of life
FDA: Food and Drug Administration
PD-1: Programmed death-1
PD-L1: Programmed death-ligand-1
CTLA-4: Cytotoxic T lymphocyte antigen 4
irAEs: Immune-related adverse effects
AACE: American Association of Clinical Endocrinology
T2DM: Type 2 diabetes mellitus
TSH: Thyroid-stimulating hormone
ACTH: Adrenocorticotropic hormone
LH: Luteinizing hormone
FSH: Follicle-stimulating hormone
PTH: Parathyroid hormone
MRI: Magnetic resonance imaging
DKA: Diabetic ketoacidosis
GAD: Glutamic acid decarboxylase
NLR: Neutrophil-to-lymphocyte ratio
PLR: Platelet-to-lymphocyte ratio
BMI: Body mass index
CKD: Chronic kidney disease
IHD: Ischemic heart disease
