# Supplementary material for: The burden and clinical trajectory of immune checkpoint inhibitor-induced endocrinopathies: an 8-year experience
Source: BMC Med. 2024 Dec 18;22:588. doi: 10.1186/s12916-024-03812-2 (PMC11656579; doi:10.1186/s12916-024-03812-2)
Supplement: Supplementary file 1 — Additional file 1: Table S1. Comprehensive pre-treatment biochemical profile of patients undergoing ICI therapy. Table S2. Dynamic changes in biochemical markers and radiological findings following ICI treatment. Table S3. End-of-study biochemical endocrine profile in patients following ICI therapy. Table S4. Clinical Characteristics of Patients Stratified by Age. Table S5. Comparison of clinical outcomes between PDL1 and PD1 inhibitors in the study population. [file 12916_2024_3812_MOESM1_ESM.docx]

| **Table S1: Comprehensive pre-treatment biochemical profile of patients undergoing ICI therapy. Data are reported as available, and reported as mean (with SD), median (with IQR), and N (with %) as appropriate.** | |
| --- | --- |
| **Parameter** | **Result (N=649)** |
| Creatinine, μmol/L | 70 (56-90) |
| Urea, mmol/L | 4.5 (3.3-6) |
| Sodium, mmol/L | 138 (135-140) |
| Potassium, mmol/L | 4.28 ± 0.51 |
| Fasting Glucose, mmol/L | 5.9 (5.1-7.5) |
| Random Glucose, mmol/L | 6.7 (5.6-9) |
| HbA1c, % | 5.85 (5.4-6.9) |
| WBC, ×10^3^/L | 6.9 (5-9.3) |
| Hemoglobin, g/dL | 11.42 (2.01) |
| Neutrophil, ×10^3^/L | 6.3 (3.7-53) |
| N/L Ratio | 3.28 (2.05-5.72) |
| Platelets, ×10^3^/L | 261 (200-353) |
| Lymphocyte Count, ×10^3^/L | 1.8 (1.2-10.3) |
| P/L Ratio | 144.44 (32.93-252.5) |
| C-Peptide, ng/mL | 0.565 (0.31-2.21) |
| TSH, mIU/L | 1.81 (1.07-3.35) |
| Free T3, pmol/L | 4.09 (1.13) |
| Free T4, pmol/L | 14.9 (12.9-16.71) |
| TRAB, IU/L | 4.95 (1.8-12.25) |
| TPO Antibodies, IU/mL | 62.5 (19-600) |
| Thyroid Enlargement (on ultrasound) | 15 (27.27%) |
| AM Cortisol, nmol/L | 349.44 (164.80) |
| ACTH, pg/mL | 9.3 (5-131) |
| FSH, IU/L | 6.2 (3.9-20.1) |
| LH, IU/L | 7.28 (4.7-19.3) |
| IGF-1, μg/L | 122.7 (40.7-210.6) |
| Testosterone, nmol/L | 21.225 (1.32-24.185) |
| Estradiol, pmol/L | 72.8 (44.4-169) |
| DHEAS, μmol/L | 2.57 (2.075-4.935) |

(IQR: Interquartile Range, SD: Standard Deviation, WBC: White Blood Cells, N/L Ratio: Neutrophil to Lymphocyte Ratio, P/L Ratio: Platelets to Lymphocyte Ratio, TSH: Thyroid Stimulating Hormone, TRAB: TSH Receptor Antibodies, TPO: Thyroid Peroxidase, AM: Morning, ACTH: Adrenocorticotropic Hormone, FSH: Follicle Stimulating Hormone, LH: Luteinizing Hormone, IGF-1: Insulin-Like Growth Factor 1, DHEAS: Dehydroepiandrosterone Sulfate). Normal ranges: Creatinine 62-106 μmol/L, Urea 2.5-7.8 mmol/L, Sodium 133-146 mmol/L, Potassium 3.5-5.3 mmol/L, Fasting Glucose <11.1 mmol/L, Random Glucose 3.3-5.5 mmol/L, HbA1c <5.7 %, WBC 4-10 ×10^3^/L, Hemoglobin 12-15 g/dL, Neutrophil 2-7 ×10^3^/L, Platelets 150-410 ×10^3^/L, Lymphocyte Count 1-3 ×10^3^/L, C-Peptide 1.1-4.4 ng/mL, TSH 0.3-4.2 mIU/L, Free T3 3.7-6.4 pmol/L, Free T4 11-23.3 pmol/L, TRAB <1.75 IU/L, TPO Antibodies 0-34 IU/mL, AM Cortisol 138-689 nmol/L, ACTH 7.2-63.3 pg/mL, FSH follicular phase, 4 - 13 IU/L; ovulation phase, 5 - 22 IU/L; luteal phase, 2 - 8 IU/L; post-menopausal phase, 26 - 135 IU/L, LH follicular phase, 2 - 13 IU/L; ovulation phase, 14 - 96 IU/L; luteal phase, 1 - 11 IU/L; post-menopausal phase, 8 - 59 IU/L, IGF-1 91-238 μg/L , Testosterone 10.4-37.4 nmol/L, Estradiol Follicular Phase, 45.4 - 854 pmol/L; Midcycle Phase, 151 - 1461 pmol/L; Luteal Phase, 81.9 - 1251 pmol/L; PMP Phase, <18.4 - 505 pmol/L, DHEAS 4.02-11 μmol/L.

| **Table S2: Dynamic changes in biochemical markers and radiological findings following ICI treatment. Data reported as available, reported as mean (with SD), median (with IQR), and N (with %) as appropriate.** | |
| --- | --- |
| **Biochemical parameters** | **Results** |
| TPO (N=36)  Newly positive  Pre-ICI positive  Negative | 7 (19.4%)  2 (5.6%)  27 (75%) |
| TRAB (N=14)  Positive  Negative | 2 (14.3%)  12 (85.7%) |
| Anti GAD (N=3)  Positive  Negative | 1 (33.3%)  2 (66.6%) |
| TSH, mIU/L | 2.4 (1.2-6.7) |
| Free T3, pmol/L | 4.2 (3.1-4.9) |
| Free T4, pmol/L | 14.4 (11.9-16.6) |
| AM Cortisol, nmol/L | 340 ± 215.8 |
| ACTH, pg/mL | 13.9 (7.3-38.5) |
| FSH, IU/L | 11.9 (4-32) |
| LH, IU/L | 8.2 (5.2-12.8) |
| IGF-1, μg/L | 128 ± 66.5 |
| Testosterone, nmol/L | 11.3 ± 8.5 |
| Prolactin, mIU/L | 284 (203-484) |
| Hypophysitis on MRI Post-ICI (N=649) | 1 (0.15%) |
| HbA1c % | 5.9 (5.4-6.8) |
| Highest Fasting Glucose | 6.9 (5.4-8.9) |
| Highest Random Glucose | 8.5 (6.9-12.1) |
| C-Peptide | 0.57 (0.3-2.2) |
| DKA (N=649) | 5 (0.8%) |
| DKA Detail (N=5)  Presenting feature of DM  During the course of pre-existing DM | 4 (80%)  1 (20%) |

(IQR: Interquartile Range, SD: Standard Deviation, TPO: Thyroid Peroxidase, TRAB: TSH Receptor Antibodies, TSH: Thyroid Stimulating Hormone, AM: Morning, ACTH: Adrenocorticotropic Hormone, FSH: Follicle Stimulating Hormone, LH: Luteinizing Hormone, IGF-1: Insulin-Like Growth Factor 1, HBA1C: Glycated hemoglobin, DKA: Diabetic ketoacidosis, DM: Diabetes mellitus). Normal ranges: Prolactin 102-495 mIU/L, Fasting Glucose <11.1 mmol/L, Random Glucose 3.3-5.5 mmol/L, HbA1c <5.7 %, C-Peptide 1.1-4.4 ng/mL, TSH 0.3-4.2 mIU/L, Free T3 3.7-6.4 pmol/L, Free T4 11-23.3 pmol/L, AM Cortisol 138-689 nmol/L, ACTH 7.2-63.3 pg/mL, FSH follicular phase, 4 - 13 IU/L; ovulation phase, 5 - 22 IU/L; luteal phase, 2 - 8 IU/L; post-menopausal phase, 26 - 135 IU/L, LH follicular phase, 2 - 13 IU/L; ovulation phase, 14 - 96 IU/L; luteal phase, 1 - 11 IU/L; post-menopausal phase, 8 - 59 IU/L, IGF-1 91-238 μg/L, Testosterone 10.4-37.4 nmol/L.

| **Table S3: End-of-study biochemical endocrine profile in patients following ICI therapy. Data are reported as available and reported as mean (with SD), median (with IQR), and N (with %) as appropriate.** | |
| --- | --- |
| **Biochemical parameters** | **Results** |
| TSH, mIU/L | 2.1 (1.1-4) |
| Free T3, pmol/L | 3.9 ± 1.3 |
| Free T4, pmol/L | 14.7 ± 3.9 |
| TRAB | 0.8 (0.8-13.5) |
| TPO | 14 (10-34) |
| AM Cortisol, nmol/L | 381 (190-514) |
| ACTH, pg/mL | 16.9 (8.7-41.2) |
| FSH, IU/L | 8.1 (4-20.4) |
| LH, IU/L | 7.4 (5.18.5) |
| IGF-1, μg/L | 97.8 (18-156) |
| Testosterone, nmol/L | 5.7 (0.6-15.3) |
| Estradiol, pmol/L | 76.4 (42.7-163.5) |
| Prolactin | 240 (182-401) |
| HbA1c % | 5.8 (5.4-6.7) |

(TSH: Thyroid Stimulating Hormone, TRAB: TSH Receptor Antibodies, TPO: Thyroid Peroxidase, AM: Morning, ACTH: Adrenocorticotropic Hormone, FSH: Follicle Stimulating Hormone, LH: Luteinizing Hormone, IGF-1: Insulin-Like Growth Factor 1, HbA1c: Hemoglobin A1c, SD: Standard Deviation, IQR: Interquartile Range). Normal ranges: Prolactin 102-495 mIU/L, TRAB <1.75 IU/L, TPO Antibodies 0-34 IU/mL, HbA1c <5.7 %, TSH 0.3-4.2 mIU/L, Free T3 3.7-6.4 pmol/L, Free T4 11-23.3 pmol/L, AM Cortisol 138-689 nmol/L, ACTH 7.2-63.3 pg/mL, FSH follicular phase, 4 - 13 IU/L; ovulation phase, 5 - 22 IU/L; luteal phase, 2 - 8 IU/L; post-menopausal phase, 26 - 135 IU/L, LH follicular phase, 2 - 13 IU/L; ovulation phase, 14 - 96 IU/L; luteal phase, 1 - 11 IU/L; post-menopausal phase, 8 - 59 IU/L, IGF-1 91-238 μg/L, Testosterone 10.4-37.4 nmol/L, Estradiol Follicular Phase, 45.4 - 854 pmol/L; Midcycle Phase, 151 - 1461 pmol/L; Luteal Phase, 81.9 - 1251 pmol/L; PMP Phase, <18.4 - 505 pmol/L.

| **Table S4: Clinical Characteristics of Patients Stratified by Age.** | | | |
| --- | --- | --- | --- |
| **Characteristics** | **Age < 60 (N=416, 64.1 %)** | **Age ≥ 60 (233, 35.9%)** | **P-Value** |
| **Gender**  Males  Females | 292 (70.2%)  124 (29.8%) | 167 (71.7%)  66 (28.3%) | 0.69 |
| **ICIs**  PD1i  PDL1i  CTLA4i | 358 (86.1%)  57 (13.7%)  1 (0.2%) | 199 (85.4%)  34 (14.6%)  0 | 0.72 |
| **IREs** | 102 (24.%) | 71 (30.4%) | 0.1 |
| **Number of IREs**  1  2  3 | N=102  86 (84.3%)  13 (12.7%)  3 (2.9%) | N=71  64 (90.1%)  4 (5.6%)  3 (4.2%) | 0.14 |
| **Mortality** | 166 (39.9%) | 116 (49.7%) | 0.01 |

ICI: Immune checkpoint inhibitor, IRE: ICI-related endocrinopathy, PD-1i: Programmed Death-1 inhibitor, PD-L1i: Programmed Death-Ligand 1 inhibitor, CTLA-4i: Cytotoxic T-Lymphocyte-Associated Protein 4 inhibitor

| **Table S5. Comparison of clinical outcomes between PDL1 and PD1 inhibitors in the study population. Data are reported as available and reported as (with SD), median (with IQR), and N (with %) as appropriate.** | | | |
| --- | --- | --- | --- |
| **Variables** | **PD-1 inhibitors**  **N=150** | **PDL-1 inhibitors**  **N=22** | **P-value** |
| Age | 57.0 ± 13.4 | 57.6 ± 8.8 | 0.8 |
| **Gender**  Female  Male | 46 (30.7%)  104 (69.3%) | 4 (18.2%)  18 (81.2%) | 0.3 |
| **Ethnicity**  Arab  South Asian  Other  White | 89 (59.3%)  45 (30%)  13 (8.7%)  3 (2%) | 14 (63.6%)  6 (27.3%)  2 (9.1%)  0 | 1 |
| **Pre-existing major endocrinopathies**  Pre-existing T2D  Pre-existing Hypothyroidism | 52 (34.7%)  12 (8%) | 11 (50%)  1 (4.6%) | 0.2  1 |
| Number of ICI doses | 9 (4-17) | 10.5 (5-24) | 0.1 |
| **Number of Endocrinopathies**  1  2  3 | 130 (86.7%)  15 (10%)  5 (3.3%) | 19 (86.4%)  2 (9.1%)  1 (4.5%) | 0.9 |
| **New IREs**  Primary Hypothyroidism  Central Hypothyroidism  Primary Hyperthyroidism  Primary Adrenal Insufficiency  Secondary Adrenal Insufficiency  Hypogonadism  GHD  Hyperprolactinemia  Diabetes Insipidus  Insulin Deficiency  Hypoparathyroidism  DKA | 95 (63.3%)  5 (3.3%)  20 (13.3%)  1 (0.7%)  12 (8%)  4 (2.7%)  2 (1.3%)  4 (2.7%)  1 (0.7%)  22 (14.7%)  9 (6%)  3 (2%) | 13 (59.1%)  0  3 (13.6%)  1 (4.6%)  1 (4.6%)  2 (9.1%)  0  1 (4.6%)  0  4 (18.2%)  1 (4.6%)  0 | 0.7  1  1  0.2  1  0.2  1  0.5  1  0.7  1  1 |
| Mortality | 64 (42.7%) | 9 (40.9%) | 0.9 |

BMI = Body Mass Index, DM = Diabetes Mellitus, GIT = Gastrointestinal Tract, GHD = Growth Hormone Deficiency, DKA = Diabetic Ketoacidosis. T2D: Type 2 diabetes mellitus, IREs: Immune checkpoint inhibitors related endocrinopathies.
